# Supplementary material for: Molecular insights into receptor binding energetics and neutralization of SARS-CoV-2 variants
Source: Nat Commun. 2021 Nov 30;12:6977. doi: 10.1038/s41467-021-27325-1 (PMC8633007; doi:10.1038/s41467-021-27325-1)
Supplement: Supplementary file 1 — Supplementary Information [file 41467_2021_27325_MOESM1_ESM.pdf]

Supplementary Information for

Molecular insights into receptor binding energetics and neutralization of SARS-CoV-2 variants

Koehler *et al*.

This PDF file includes:

- Supplementary Figures 1 to 6
- Supplementary Tables 1 to 8

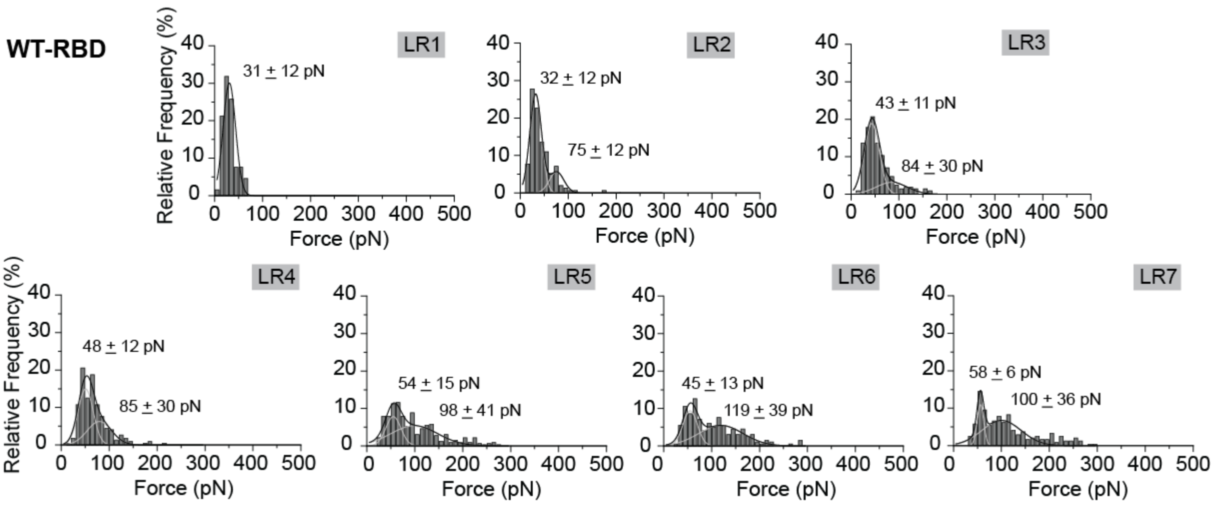

Supplementary Figure 1| Probing WT-RBD binding to ACE2 model surfaces. Force and LR were extracted from force-distance curves and sorted in narrow LR ranges (LR1-LR7). The rupture forces for each LR range were plotted as histograms and fitted with multipeak Gaussian fits. The maxima of all force peaks are indicated. N = 1435 from 4 independent experiments.

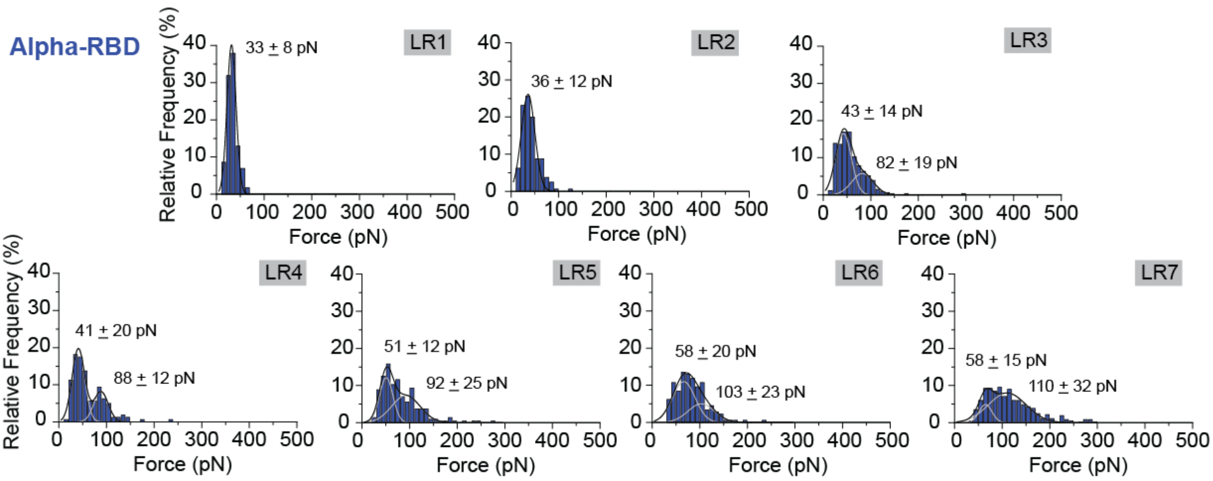

Supplementary Figure 2| Probing Alpha-RBD binding to ACE2 model surfaces. Force and LR were extracted from force-distance curves and sorted in narrow LR ranges (LR1-LR7). The rupture forces for each LR range were plotted as histograms and fitted with multipeak Gaussian fits. The maxima of all force peaks are indicated. N = 1542 from 3 independent experiments.

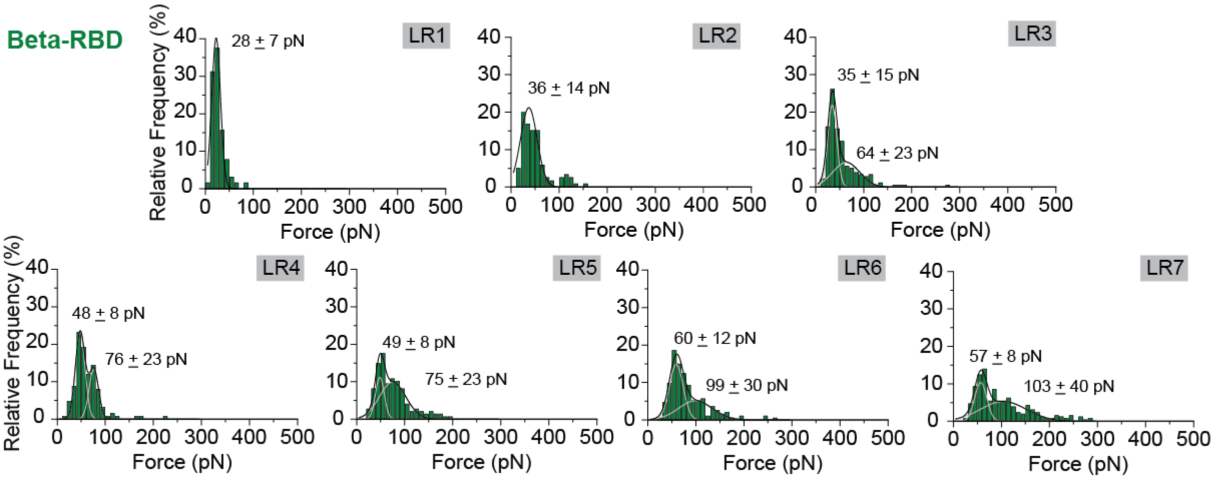

Supplementary Figure 3| Probing Beta-RBD binding to ACE2 model surfaces. Force and LR were extracted from force-distance curves and sorted in narrow LR ranges (LR1-LR7). The rupture forces for each LR range were plotted as histograms and fitted with multipeak Gaussian fits. The maxima of all force peaks are indicated. N = 1095 from 3 independent experiments.

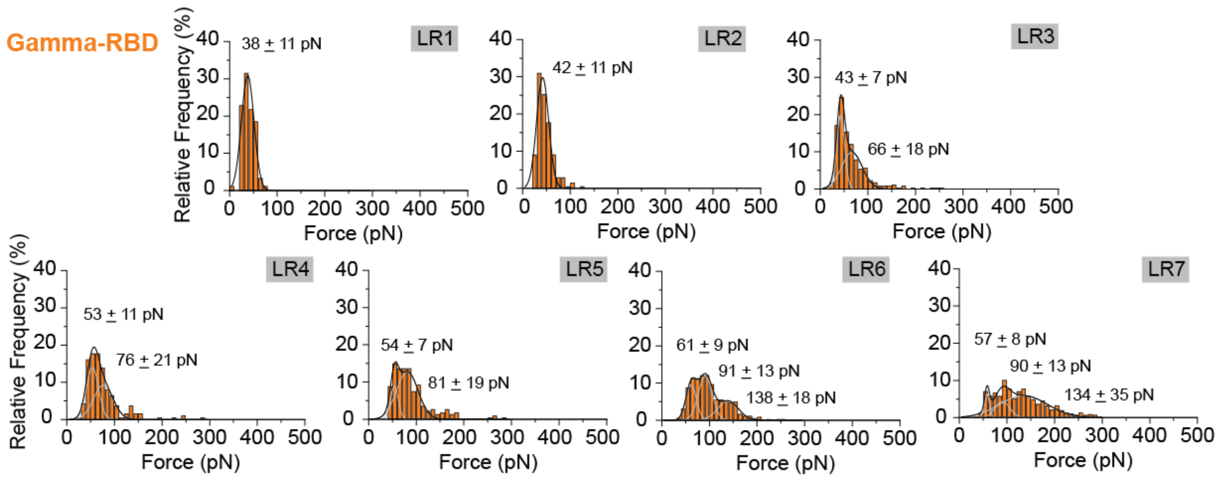

Supplementary Figure 4| Probing Gamma-RBD binding to ACE2 model surfaces. Force and LR were extracted from force-distance curves and sorted in narrow LR ranges (LR1-LR7). The rupture forces for each LR range were plotted as histograms and fitted with multipeak Gaussian fits. The maxima of all force peaks are indicated. N = 1953 from 4 independent experiments.

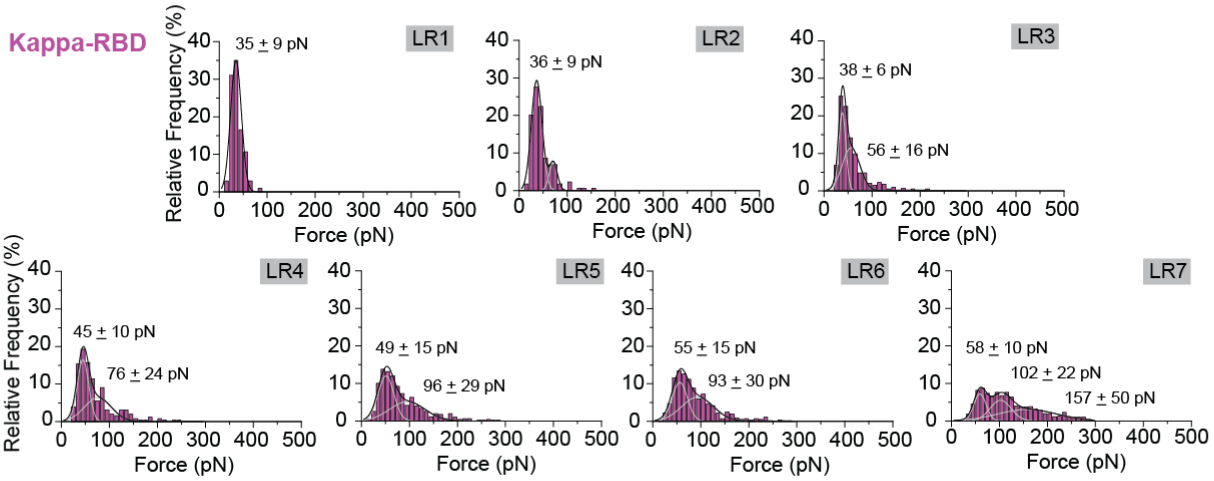

Supplementary Figure 5| Probing Kappa-RBD binding to ACE2 model surfaces. Force and LR were extracted from force-distance curves and sorted in narrow LR ranges (LR1-LR7). The rupture forces for each LR range were plotted as histograms and fitted with multipeak Gaussian fits. The maxima of all force peaks are indicated. N = 2720 from 4 independent experiments.

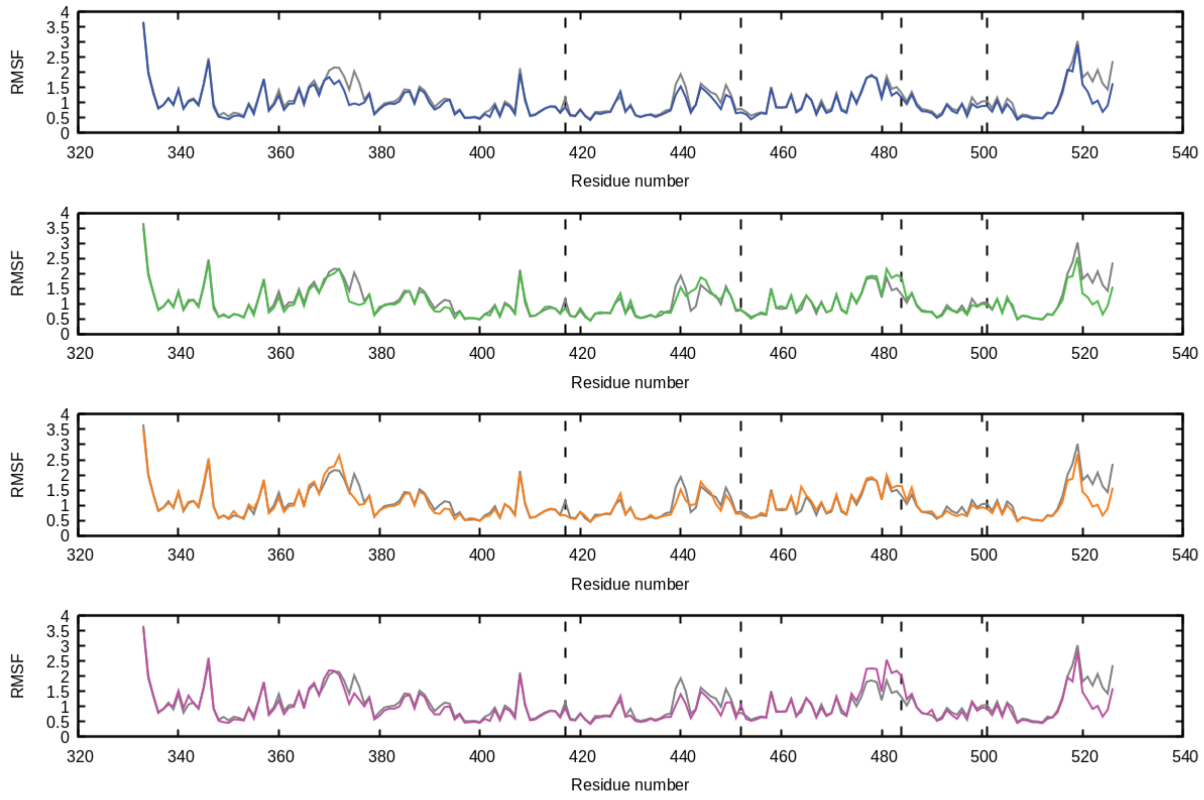

Supplementary Figure 6| RMSF calculated in the RBD region from MD trajectories for WT and variants. This plot reveals small differences in overall stiffness of the spike protein RBD. Vertical dashed lines indicate the position of the residue 417, 452, 484 and 501. Color convention as in the main manuscript: WT-RBD (grey), Alpha-RBD (blue), Beta-RBD (green), Gamma-RBD (orange) and Kappa-RBD (purle). Error bar is given by the thickness of the line.

Supplementary Table 1| Comparison P-values of K_D for the interaction between ACE2 and RBD–VoCs. According to the NEJM (New England Journal of Medicine) statistics style, P-values above 5E-2 (0.05) were considered as statistically not significant (highlighted in red), whereas P-values blow/equal 5E-2 (0.05) were considered as significant and P-values below/ equal 5E-1 (0.01, highlighted in yellow) were considered significant obviously (highlighted in green). P-values were determined by two-sample t-test in Origin.

**WT-RBD** 134 ± 81 nM	**Alpha-RBD** 129 ± 81 nM	**Beta-RBD** 80 ± 49 nM	**Gamma-RBD** 21 ± 16 nM	**Kappa-RBD** 71 ± 31 nM	
X	9.1E-1	7.4E-2	1.4E-5	1.9E-2	**WT-RBD** 134 ± 81 nM
	X	1.0E-1	3.5E-5	3.4E-2	**Alpha-RBD** 129 ± 81 nM
		X	2.9E-4	6.2E-1	**Beta-RBD** 80 ± 49 nM
			X	5.8E-6	**Gamma-RBD** 21 ± 16 nM
				X	**Kappa-RBD** 71 ± 31 nM

NEJM-Style:
ns: P > 5E-2
* P ≤ 5E-2
** P ≤ 1E-2
*** P ≤ 1E-3

Supplementary Table 2| High frequency contacts (Freq >0.7) established at the RBD/ACE2 interface in the WT. The type of interaction between residue(ACE2)-residue(RBD) contact is highlighted as follows: salt bridge -SB (in yellow), polar -PP (green) and hydrophobic -HH (blue) interaction. Fraction of hydrogen bond (HB) for each contact (if available) is calculated from the molecular trajectory.

ACE2		RBD		Freq	Type	HB
ASP (-)	30	LYS (+)	417	0.712	SB	0.386
GLN	24	GLY	476	0.790	NO	-
TYR	83	ASN	487	1.000	NO	0.371
GLU (-)	35	GLN	493	0.891	NO	0.186
ARG (+)	357	THR	500	0.890	NO	-
GLU (-)	37	TYR	505	0.781	NO	0.175
LYS (+)	31	PHE	456	0.947	NO	-
THR	27	TYR	489	0.975	NO	-
ASN	330	THR	500	0.895	PP	0.002
THR	27	ALA	475	0.930	NO	-
HIS (+)	34	LEU	455	0.960	NO	-
LYS	31	LEU	455	0.812	NO	-
LYS (+)	353	GLY	502	0.952	NO	0.400
TYR	41	GLN	498	0.924	NO	0.002
HIS (+)	34	GLN	493	0.824	NO	0.001
THR	27	TYR	473	0.904	NO	-
GLN	24	ASN	487	0.960	PP	0.021
ASP (-)	355	THR	500	0.870	NO	0.310
TYR	83	PHE	486	0.960	HH	-
LYS (+)	353	ASN	501	1.000	NO	0.001
PHE	28	TYR	489	1.000	HH	-
LYS (+)	31	GLU (-)	484	0.770	SB	0.034
ARG (+)	393	TYR	505	0.707	NO	-
THR	27	PHE	456	1.000	NO	-
LYS (+)	31	GLN	493	0.820	NO	0.062
MET	82	PHE	486	1.000	HH	-
LEU	79	PHE	486	0.970	HH	-
GLN	24	ALA	475	0.924	NO	0.058
ASP (-)	30	PHE	456	0.894	NO	-
LYS (+)	31	TYR	489	1.000	NO	-
GLY	354	GLY	502	0.970	NO	0.005
HIS (+)	34	TYR	453	0.960	NO	0.042
LYS (+)	353	TYR	505	1.000	NO	-
TYR	41	ASN	501	0.793	NO	-
TYR	41	THR	500	0.964	NO	-

Supplementary Table 3| High frequency contacts (Freq >0.7) established at the RBD/ACE2 interface in the Alpha variant. The type of interaction between residue(ACE2)-residue(RBD) contact is highlighted as follows: salt bridge -SB (in yellow), polar -PP (green) and hydrophobic -HH (blue) interaction. Fraction of hydrogen bond (HB) for each contact (if available) is calculated from the molecular trajectory.

ACE2		RBD		Freq	Type	HB
ASP (-)	30	LYS (+)	417	0.890	SB	0.410
GLN	24	GLY	476	0.790	NO	-
TYR	83	ASN	487	0.992	NO	0.353
GLY	354	TYR	505	0.752	NO	-
ARG (+)	357	THR	500	0.930	NO	-
LYS (+)	31	PHE	456	0.94	NO	-
HIS (+)	34	TYR	453	0.960	NO	0.030
ASN	330	THR	500	0.920	PP	-
THR	27	ALA	475	0.940	NO	-
HIS (+)	34	LEU	455	0.972	NO	-
LYS (+)	31	LEU	455	0.760	NO	-
LYS (+)	353	GLY	502	1.000	NO	0.532
TYR	41	GLN	498	0.905	NO	-
HIS (+)	34	GLN	493	0.820	NO	0.002
THR	27	TYR	473	0.940	NO	-
GLN	24	ASN	487	0.970	PP	0.020
ASP (-)	355	THR	500	0.910	NO	0.360
TYR	83	PHE	486	0.980	HH	-
ASP (-)	30	PHE	456	0.930	NO	-
PHE	28	TYR	489	0.980	HH	-
LYS (+)	31	GLU (-)	484	0.770	SB	0.020
ARG (+)	393	TYR	505	0.750	NO	0.001
THR	27	PHE	456	1.000	NO	-
LYS (+)	31	TYR	489	1.000	NO	-
LYS (+)	31	GLN	493	0.770	NO	0.050
MET	82	PHE	486	1.000	HH	-
LEU	79	PHE	486	0.973	HH	-
GLN	24	ALA	475	0.930	NO	0.030
LYS (+)	353	TYR	501	1.000	NO	0.002
THR	27	TYR	489	0.983	NO	0.001
GLY	354	GLY	502	0.972	NO	-
TYR	41	TYR	501	0.940	HH	-
LYS (+)	353	TYR	505	1.000	NO	-
GLU (-)	35	GLN	493	0.790	NO	0.141
TYR	41	THR	500	0.953	NO	0.001

Supplementary Table 4| High frequency contacts (Freq >0.7) established at the RBD/ACE2 interface in the Beta variant. The type of interaction between residue(ACE2)-residue(RBD) contact is highlighted as follows: salt bridge -SB (in yellow), polar -PP (green) and hydrophobic -HH (blue) interaction. Fraction of hydrogen bond (HB) for each contact (if available) is calculated from the molecular trajectory.

ACE2		RBD		Freq	Type	HB
LYS (+)	353	GLY	502	1.000	NO	0.470
LYS (+)	31	TYR	489	1.000	NO	-
GLN	24	GLY	476	0.810	NO	-
HIS (+)	34	LEU	455	0.830	NO	-
TYR	83	ASN	487	1.000	NO	0.001
HIS (+)	34	GLN	493	0.920	NO	0.001
GLU (-)	35	GLN	493	0.870	NO	0.160
GLY	354	GLY	502	0.941	NO	0.007
ASP (-)	355	THR	500	0.890	NO	0.350
ASP (-)	30	PHE	456	0.830	NO	-
LYS (+)	31	PHE	456	0.924	NO	-
TYR	83	PHE	486	0.971	HH	-
TYR	41	TYR	501	0.970	HH	0.001
THR	27	TYR	489	0.980	NO	-
GLN	24	ASN	487	0.970	PP	0.021
PHE	28	TYR	489	0.981	HH	-
HIS (+)	34	TYR	453	0.960	NO	0.053
ASN	330	THR	500	0.920	PP	-
THR	27	PHE	456	1.000	NO	-
THR	27	ALA	475	0.940	NO	-
LYS (+)	31	GLN	493	0.784	NO	0.113
MET	82	PHE	486	0.992	HH	-
LEU	79	PHE	486	0.964	HH	-
GLN	24	ALA	475	0.930	NO	0.044
LYS (+)	353	TYR	501	1.000	NO	0.010
ARG (+)	357	THR	500	0.872	NO	-
LYS (+)	31	LEU	455	0.800	NO	-
THR	27	TYR	473	0.890	NO	-
LYS (+)	353	TYR	505	0.994	NO	-
TYR	41	GLN	498	0.890	NO	-
TYR	41	THR	500	0.980	NO	0.003

Supplementary Table 5| High frequency contacts (Freq >0.7) established at the RBD/ACE2 interface in the Gamma variant. The type of interaction between residue(ACE2)-residue(RBD) contact is highlighted as follows: salt bridge -SB (in yellow), polar -PP (green) and hydrophobic -HH (blue) interaction. Fraction of hydrogen bond (HB) for each contact (if available) is calculated from the molecular trajectory.

ACE2		RBD		Freq	Type	HB
GLN	24	GLY	476	0.824	NO	-
TYR	83	ASN	487	0.990	NO	0.001
GLU	35	GLN	493	0.930	NO	0.210
ARG	357	THR	500	0.882	NO	0.001
GLU (-)	37	TYR	505	0.750	NO	0.113
LYS (+)	31	PHE	456	0.934	NO	-
HIS (+)	34	TYR	453	0.960	NO	0.007
ASN	330	THR	500	0.890	PP	0.002
THR	27	ALA	475	0.930	NO	-
ASP	30	LEU	455	0.810	NO	-
HIS (+)	34	LEU	455	0.994	NO	-
LYS (+)	31	LEU	455	0.714	NO	-
LYS (+)	353	GLY	502	0.950	NO	0.461
LYS (+)	31	TYR	489	1.000	NO	-
HIS (+)	34	GLN	493	0.800	NO	0.001
THR	27	TYR	473	0.900	NO	-
GLY	354	GLY	502	0.840	NO	-
ASP (-)	355	THR	500	0.760	NO	0.284
TYR	83	PHE	486	0.900	HH	-
PHE	28	TYR	489	0.982	HH	-
ASP (-)	30	PHE	456	0.864	NO	-*
TYR	41	GLN	498	0.870	NO	0.002
THR	27	PHE	456	1.000	NO	-
THR	27	TYR	489	0.980	NO	-
LYS (+)	31	GLN	493	0.834	NO	0.120
MET	82	PHE	486	0.950	HH	-
LEU	79	PHE	486	0.912	HH	-
GLN	24	ALA	475	0.922	NO	0.050
LYS (+)	353	TYR	501	1.000	NO	0.010
TYR	83	TYR	489	0.702	HH	0.010
TYR	41	TYR	501	0.980	HH	-
GLY	354	TYR	505	0.763	NO	-
LYS (+)	353	TYR	505	1.000	NO	0.001
GLN	24	ASN	487	0.961	PP	0.021
TYR	41	THR	500	0.990	NO	0.001

Supplementary Table 6| High frequency contacts (Freq >0.7) established at the RBD/ACE2 interface in the Kappa variant. The type of interaction between residue(ACE2)-residue(RBD) contact is highlighted as follows: salt bridge -SB (in yellow), polar -PP (green) and hydrophobic -HH (blue) interaction. Fraction of hydrogen bond (HB) for each contact (if available) is calculated from the molecular trajectory.

ACE2		RBD		Freq	Type	HB
ASP (-)	30	LYS (+)	417	0.850	SB	0.410
GLN	24	GLY	476	0.750	NO	-
TYR	83	ASN	487	0.992	NO	0.373
GLU (-)	35	GLN	493	0.940	NO	0.210
ARG (+)	357	THR	500	0.870	NO	-
GLU (-)	37	TYR	505	0.750	NO	0.241
LYS (+)	31	PHE	456	0.950	NO	-
THR	27	TYR	489	0.984	NO	0.002
ASN	330	THR	500	0.872	PP	-
THR	27	ALA	475	0.870	NO	-
GLN	42	GLN	498	0.704	PP	0.060
HIS (+)	34	LEU	455	0.960	NO	-
LYS (+)	31	LEU	455	0.862	NO	-
LYS (+)	353	GLY	502	0.980	NO	0.470
TYR	41	GLN	498	0.950	NO	0.001
HIS (+)	34	GLN	493	0.830	NO	0.002
THR	27	TYR	473	0.910	NO	-
GLN	24	ASN	487	0.970	PP	0.020
ASP (-)	355	THR	500	0.852	NO	0.320
TYR	83	PHE	486	0.970	HH	-
LYS (+)	353	ASN	501	1.000	NO	0.001
PHE	28	TYR	489	0.980	HH	-
THR	27	PHE	456	1.000	NO	-
LYS (+)	31	GLN	493	0.812	NO	0.070
MET	82	PHE	486	1.000	HH	-
LEU	79	PHE	486	1.000	HH	-
GLN	24	ALA	475	0.920	NO	0.040
ASP (-)	30	PHE	456	0.900	NO	-
LYS (+)	31	TYR	489	1.000	NO	-
HIS (+)	34	TYR	453	0.960	NO	0.010
LYS (+)	353	TYR	505	1.000	NO	0.030
GLY	354	GLY	502	0.950	NO	0.001
TYR	41	THR	500	1.000	NO	0.002

Supplementary Table 7| All contacts established at the RBD/ACE2 interface for residues involved in mutation (i.e. 417, 484, 452 and 501). The type of interaction between residue(ACE2)-residue(RBD) contact is highlighted as follows: salt bridge -SB (in yellow), polar -PP (green) and hydrophobic -HH (blue) interaction. The distance (d_{CA-CA}) and fluctuation ($\delta(d_{CA-CA})$) for a given contact is provided.

	ACE2		RBD		Freq	d_{CA} [Å]	$\delta(d_{CA})$ [Å²]	Type
**residue 417**								
WT	APS(-)	30	LYS(+)	417	0.712	11.310	0.421	SB
Alpha	APS(-)	30	LYS(+)	417	0.890	11.553	0.560	SB
Beta	APS(-)	30	ASN	417	0.300	11.531	0.267	NO
Gamma	HIS(+)	34	THR	417	0.360	11.799	0.188	NO
Kappa	APS(-)	30	LYS(+)	417	0.850	11.450	0.510	SB
**residue 484**								
WT	LYS(+)	31	GLU(-)	484	0.770	14.483	0.248	SB
Alpha	LYS(+)	31	GLU(-)	484	0.771	14.526	0.235	SB
Beta	GLU(-)	75	LYS(+)	484	0.220	14.266	1.553	SB
Gamma	GLU(-)	75	LYS(+)	484	0.240	14.037	1.648	SB
Kappa	LYS(+)	31	GLN	484	0.300	14.052	1.288	NO
**residue 501**								
WT	LYS(+)	353	ASN	501	0.997	9.147	0.132	NO
	TYR	41	ASN	501	0.793	11.269	0.307	NO
Alpha	LYS(+)	353	TYR	501	1.000	9.132	0.072	NO
	APS(-)	355	TYR	501	0.241	6.612	0.204	NO
	APS(-)	38	TYR	501	0.569	12.985	0.275	NO
	TYR	41	TYR	501	0.940	11.778	0.270	HH
	GLN	42	TYR	501	0.190	11.502	0.312	NO
Beta	GLY	352	TYR	501	0.166	9.521	0.260	NO
	LYS(+)	353	TYR	501	1.000	9.097	0.133	NO
	APS(-)	355	TYR	501	0.316	6.385	0.256	NO
	APS(-)	38	TYR	501	0.556	12.730	0.277	NO
	TYR	41	TYR	501	0.969	11.606	0.268	HH
	GLN	42	TYR	501	0.172	11.331	0.346	NO
Gamma	LYS(+)	353	TYR	501	0.999	9.419	0.450	NO
	APS(-)	355	TYR	501	0.142	7.016	0.419	NO
	APS(-)	38	TYR	501	0.401	12.789	0.325	NO
	TYR	41	TYR	501	0.978	11.579	0.299	HH
	GLN	42	TYR	501	0.272	11.423	0.321	NO
Kappa	LYS(+)	353	ASN	501	0.995	9.118	0.166	NO
	APS(-)	355	ASN	501	0.332	6.910	0.220	NO
	TYR	41	ASN	501	0.638	11.322	0.325	NO

Supplementary Table 8| Intra-chain contacts established by residue 452 in RBD. The type of interaction between residue(RBD)-residue(RBD) contact is highlighted as follows: salt bridge - SB (in yellow), polar -PP (green) and hydrophobic -HH (blue) interaction. The distance (d_{CA-CA}) and fluctuation ($\delta(d_{CA-CA})$) for a given contact are provided.

	RBD		RBD		Freq	d_{CA} [Å]	$\delta(d_{CA})$ [Å²]	Type
WT	SER	349	LEU	452	0.999	5.034	0.118	NO
	VAL	350	LEU	452	0.794	4.339	0.128	HH
	TYR	351	LEU	452	0.997	5.193	0.221	HH
	LEU	452	TYR	495	0.393	4.696	0.060	HH
	LEU	452	PHE	490	0.900	14.894	0.199	HH
	LEU	452	SER	494	0.992	7.474	0.074	NO
	LEU	452	LEU	492	0.994	9.829	0.117	HH
	LEU	452	GLN	493	1.000	6.880	0.079	NO
Alpha	SER	349	LEU	452	1.000	4.915	0.076	NO
	VAL	350	LEU	452	0.972	4.256	0.099	HH
	TYR	351	LEU	452	0.998	5.083	0.097	HH
	LEU	452	TYR	495	0.237	4.715	0.074	HH
	LEU	452	PHE	490	0.889	14.985	0.151	HH
	LEU	452	LEU	492	0.992	9.913	0.101	HH
	LEU	452	SER	494	0.998	7.480	0.073	NO
	LEU	452	GLN	493	1.000	6.931	0.073	NO
Beta	SER	349	LEU	452	0.996	5.113	0.216	NO
	VAL	350	LEU	452	0.781	4.278	0.133	HH
	TYR	351	LEU	452	0.972	5.134	0.229	HH
	LEU	452	TYR	495	0.401	4.780	0.105	HH
	LEU	452	PHE	490	0.888	14.835	0.238	HH
	LEU	452	SER	494	0.954	7.466	0.084	NO
	LEU	452	GLN	493	0.968	6.867	0.085	NO
	LEU	452	LEU	492	0.996	9.845	0.138	HH
Kappa	SER	349	ARG(+)	452	1.000	4.980	0.089	NO
	VAL	350	ARG(+)	452	0.963	4.201	0.096	NO
	TYR	351	ARG(+)	452	0.999	5.114	0.109	NO
	ARG(+)	452	TYR	495	0.271	4.655	0.036	NO
	ARG(+)	452	PHE	490	0.343	14.876	0.180	NO
	ARG(+)	452	LEU	492	0.942	9.985	0.110	NO
	ARG(+)	452	SER	494	0.999	7.474	0.055	NO
	ARG(+)	452	GLN	493	1.000	6.935	0.074	NO
Gamma	SER	349	LEU	452	1.000	4.951	0.110	NO
	VAL	350	LEU	452	0.849	4.386	0.177	HH
	TYR	351	LEU	452	0.993	5.161	0.193	HH
	LEU	452	THR	470	0.130	12.559	0.209	NO
	LEU	452	TYR	495	0.267	4.733	0.044	HH

	LEU	452	PHE	490	0.894	14.918	0.178	HH
	LEU	452	SER	494	0.996	7.469	0.067	NO
	LEU	452	LEU	492	0.997	9.832	0.110	HH
	LEU	452	GLN	493	1.000	6.946	0.075	NO
